# AFFECT VARIABILITY AND SLEEP: EMOTIONAL UPS AND DOWNS ARE RELATED TO A POORER NIGHT’S REST

**DOI:** 10.1093/geroni/igz038.2714

**Published:** 2019-11-08

**Authors:** Kate A Leger, Susan Charles, Karen Fingerman

**Affiliations:** 1 University of Kentucky, Lexington, Kentucky, United States; 2 UC Irvine, Irvine, California, United States; 3 University of Texas Austin, Austin, Texas, United States

## Abstract

Emotional experience is strongly related to physical health. Yet, fluctuations in daily emotional experience, known as affect variability, have been less examined. It is unknown how affect variability throughout the day is related to sleep, a critical health behavior. The current study examines this relationship in an ecological momentary assessment of 277 older adults. Regression models indicate that greater variability in daily positive affect is associated with fewer hours of sleep (b = -0.648, p = .04) and greater morning tiredness (b = 0.67, p = .006) even after adjusting for mean levels of affect. Although greater negative affect variability is associated with worse sleep quality (b = -0.77, p = .02) and greater morning tiredness (b = 0.91, p = .004), these relationships disappear once mean negative affect is added into the model. Findings support models describing the downside in the fragility of positive affect.

